# Supplementary material for: Domain classification of archaeal proteomes reveals conserved fold repertoire
Source: PLoS Comput Biol. 2026 Sep 18;22(9):e1014188. doi: 10.1371/journal.pcbi.1014188 (PMC13607799; doi:10.1371/journal.pcbi.1014188)
Supplement: Supplementary file 2 [file pcbi.1014188.s007.docx]

**Response to reviewers for PCOMPBIOL-D-26-00716R1**

We thank the reviewers for their prompt and informative comments. Specifically, with respect to R2’s difficulty in accessing the two supplementary items: 1) the novel folds tarball and 2) the SI Table 1, these items were added in revision and were not in our initial Zenodo repository. We found and corrected a stale link in the SI Table legend that referred to our pre-submission repo (now 10.5281/zenodo.21656443). We also changed the Zenodo repo to preview the Readme (containing the file listing) on load. The S1 Table appears in both the manuscript and the Zenodo repo; the novel fold structures are sufficiently large that they can only appear in the Zenodo repo. We hope this change gives the reviewer clear access, if they are still having trouble accessing the data we’ll need more information on the route they are using.

<https://zenodo.org/records/21656443/files/S1_Table.tsv.gz?download=1>
https://zenodo.org/records/21656443/files/archaea_novel_fold_structures_cif.tar.gz?download=1
