# Supplementary material for: Domain classification of archaeal proteomes reveals conserved fold repertoire
Source: PLoS Comput Biol. 2026 Sep 18;22(9):e1014188. doi: 10.1371/journal.pcbi.1014188 (PMC13607799; doi:10.1371/journal.pcbi.1014188)
Supplement: Supplementary file 4 [file pcbi.1014188.s006.docx]

## To the Academic Editor and Reviewers

We thank you for the constructive review and are grateful for the reviewers' positive assessments. We have revised the manuscript substantially. The principal additions are:

1. **A compiled, single-table supplementary data product** (Reviewer 2): one joined master table of all 204,758 classified domains (36 columns) containing every field Reviewer 2 requested : identifier, organism, Pfam, detected fold, H-group/X-group, the four Foldseek/MMseqs2 clusters (PSC, PXC, DSC, DXC), mean pLDDT, and structure origin, so the full result set can be used without joining across files.
2. **A global workflow schematic (new Fig 1A)** (Reviewer 2).
3. **A deposited collection of the 2,852 candidate novel-fold structures** with a per-domain summary table (Reviewer 2).
4. **A phylogenetic mapping of the fold repertoire onto the six major groups (S2 Fig)** (Reviewer 1.3) and a **sequence-based disorder analysis of the unclassified fraction (S3 Fig)** (Reviewer 1.7).
5. **An "Implications for eukaryotic origins" Discussion section** that shares our reading of the two-domain question (Reviewer 1.8).
6. **Re-verification of all ECOD-derived counts against a frozen snapshot of ECOD v292**; a few taxonomic-occupancy numbers were updated on revision to match the current source (noted where relevant).

# Reviewer #1

*We thank the reviewer for the generous summary ("informative, well written … in-depth analysis of the sequence to structure anatomy of proteins") and address each concern below.*

**1. Clarification of Abstract Phrasing.**

*I am confused by the abstract sentence: "40% of known structural diversity within a single domain of life." Does this imply that this single archaeal dataset encompasses 40% of the diversity found across all structural domains? Please clarify.*

We have clarified the ambiguity in the original statement. The Abstract now states that archaeal proteins span 987 of the 2,457 ECOD X-groups defined across *all cellular life*; i.e., roughly 40% of the X-groups known in the structural universe have at least one representative within this single domain of life. The same explicit framing is used at first mention in the Results.

**2. Structure Quality Metrics.**

*On page 7, the text refers to proteins that "have structure quality metrics." Please specify which exact metrics (e.g., pLDDT, PAE) or thresholds were utilized to evaluate model confidence.*

We now specify the metrics and thresholds. Per-residue pLDDT (defined at first use) is the primary confidence metric; PAE is computed and retained for all de novo predictions. Thresholds now stated in text: pLDDT < 70 for the low-confidence cutoff in the dark-matter analysis, pLDDT < 50 to flag disordered residues, and a DPAM probability of 0.85 for the "high-confidence"/"well-assigned" domain category. These appear in Results ("Dataset composition"; dark-matter section) and Methods.

**3. Phylogenetic Tree of Protein Classes.**

*A phylogenetic tree of the 6 main classes of archaeal proteins would significantly enhance the reader's grasp of the evolutionary trajectory of these architectures.*

We have added **S2 Fig**: a cladogram of the six major archaeal groups with each group's high-confidence H-group repertoire mapped onto the tips (universal core present in all six / shared by two–five groups / group-specific). A new paragraph ("Domain repertoire across archaeal lineages") reports the trajectory: a universal core of 551 of 1,583 H-groups (35%) underlies all six groups and forms the majority of each group's repertoire (from ~72% in reduced lineages to ~45–52% in larger ones), so repertoire size tracks lineage through shared accessory folds rather than new fold classes.

**4. Distinction of Asgard-Specific Distributions.**

*…"The distinction between Asgard-specific and pan-archaeal distribution has implications that are beyond the scope of this study but merit investigation…" A brief hint about what type of distinction was observed is highly warranted.*

We now characterize the distinction concretely through the major vault protein (MVP) case study (Fig 8): although MVP was recently proposed as an Asgard–eukaryote structural connection, our pan-archaeal classification finds 32 high-confidence MVP domains across all six major groups, with Asgard accounting for only 5 (16%) (i.e., a feature that appears Asgard-specific under sparse sampling is in fact pan-archaeal). The contrasting lineage-restricted case (MCR, archaea-exclusive and methanogen-associated) is shown alongside it, and the point is developed in the new "Implications for eukaryotic origins" section.

**5. Methods Reference for Topology.**

*…ensure you directly point the reader to the Methods section for explicit definitions of terms like "Partial Domain" or "simple topology."*

Done, a "see Methods" pointer is added at first use of the DPAM judge categories in Results, and Methods now defines each category ("good domain," "simple topology," "low confidence," "partial domain") in plain language.

**6. Figure 8 Caption Discrepancies.**

*The caption for Figure 8 does not make sense. Panel (A) appears to refer to structures in (B) and vice versa, and some structures are omitted entirely. It is unclear what the reader should be looking for in display (B); the labels require untangling.*

Figure 8 has been rebuilt as a coherent three-panel figure with a corrected, fully specified caption: (A) pan-archaeal MVP across five of the six groups; (B) lineage-restricted MCR; (C) the eukaryotic MVP reference (rat-liver vault, PDB 2ZUO). The panel↔structure correspondence, the per-panel domain colour scheme, and every displayed accession are now stated explicitly.

**7. Evaluation of Disordered Proteins.**

*…sequences "are predominantly disordered or poorly predicted." Since these proteins might hide structural novelty, disordered proteins or regions could be explicitly evaluated using sequence-based disorder predictors.*

**S3 Fig** and a new results paragraph report a sequence-based disorder analysis (metapredict V3, independent of AlphaFold) of the 8,760 domain-free proteins against a high-confidence classified control. The domain-free fraction is a mixture of genuine intrinsic disorder (~24% predominantly disordered, essentially absent from the classified control), short/low-confidence proteins, and a small ordered-but-low-confidence residual — not concealed novelty. Sequence disorder correlates only weakly with structure confidence (r = −0.19), showing "disordered" and "poorly predicted" are separable; and the curated novel-fold candidates are almost entirely sequence-ordered (288 of 295), confirming they are genuine folds.

**8. Woese vs. two-domain?**

*This analysis should inform the two-domain model, and I am disappointed that the authors do not share their insight on this question, especially given its apparent relevance.*

We appreciate this prompt and have added a dedicated Discussion section, "Implications for eukaryotic origins," that states our view. At the single-domain level we recover a substantial archaeal–eukaryotic shared repertoire,143 H-groups shared by archaea and eukaryotes but absent from bacteria (118 with a confidently assigned eukaryotic homolog), including the recognizable information-processing core (archaeal-/eukaryotic-specific RNA-polymerase subunits such as Rpb5 and Rpb10, tRNA-splicing endonucleases, replication and translation factors) expected of a shared informational heritage, now resolved across all major archaeal lineages rather than a single model archaeon. We share the insight that this is consistent with an archaeal ancestry of the eukaryotic informational machinery, while being explicit that fold-occupancy data alone do not adjudicate between the two- and three-domain models: we show, for example, that several folds proposed as Asgard–eukaryote connections (most clearly MVP) are in fact pan-archaeal, so we caution against reading Asgard–eukaryote fold-sharing directly as phylogenetic signal. Definitive resolution requires deeper Asgard sampling than any current whole-proteome set provides, which we identify as the natural next step.

**9. Data accessibility and structure.**

*…provide a clearer description, possibly in the supplemental materials, of how the data are structured… Specifically, is the dataset searchable? Are bulk download options available?*

The "Data and code availability" section now states plainly that (i) the complete per-domain classification is available as a single downloadable table (CC-BY-4.0) and the full frozen exports are at Zenodo (bulk download), and (ii) the predicted structures and domain assignments are additionally **searchable** at the EPP resource (<http://prodata.swmed.edu/ecod2/epp>). The repository structure (the compiled master table plus its column dictionary) is described in the same section and in the deposit README.

# Reviewer #2

*We thank the reviewer for the careful reading and for the specific, actionable suggestions, which have materially improved the manuscript's clarity and its usefulness to the community.*

## General comments

*The absence of line numbers complicates the review process.*

Line numbers have been added to the revised manuscript.

*A global schematic summarizing the workflow of different datasets used in the study is missing…*

Added as **Fig 1A**: sampling (GTDB r220) → structure provenance (pre-existing AFDB vs. de novo AlphaFold3) → DPAM/ECOD classification → Pfam + four-level clustering → dark-matter funnel, with the study's original contributions highlighted by a provenance colour key.

*The results should be compiled into supplementary material, including at least identifiers, associated characteristics (organism, Pfam domains, detected folds, H-group/X-group, Foldseek/MMseqs2 clusters (PSC, PXC, DSC, DXC), average pLDDT scores, origin of the structure). Additionally, a collection of predicted structures with unknown folds would be highly valuable.*

Both are now provided. (i) A single joined **master table** of all 204,758 domains contains every field listed: identifier, organism, Pfam, detected fold, H-group and X-group, all four cluster memberships (PSC, PXC, DSC, DXC), mean pLDDT, and structure origin (pre-existing AFDB vs. de novo AF3), with a column dictionary. (ii) A discrete **novel-fold collection** of the 2,852 candidate dark-matter structures (mmCIF) is deposited with a per-domain summary (cross-reference, chopping, residue count, pLDDT, structural cluster, provisional label). Both are added to the deposit and cited in Data and code availability.

## Specific comments

*"Proteins were drawn" — specify "protein structures".*

Reworded; the source description now refers to protein structures (the AFDB contribution is described as pre-existing structures).

*"From the AFDB" — clarify "from AlphaFold2 (AF2)".*

Clarified: the AFDB-sourced structures are pre-existing AlphaFold2 predictions. We additionally reframed the whole axis as structure **provenance**: "pre-existing (AFDB)" vs. "newly predicted in this study (AlphaFold3)", which we found clearer than AF2/AF3 alone. The AF2/AF3 method labels are retained where the comparison is specifically between prediction methods (S1 Fig).

*Define terms such as pLDDT, DPAM judge, T-group, etc.*

All are now defined at first use: pLDDT (per-residue confidence, 0–100), the DPAM judge categories (with a Methods pointer), T-group (topology group within a homologous H-group), and "sub-threshold" (see below).

*"DPAM assigned 204,758" — total number of redundant domains? What about non-redundant?*

Clarified: 204,758 is the full (redundant) domain count across 115,623 proteins; on clustering these reduce to 123,169 domain sequence clusters (MMseqs2) and 52,341 domain structural clusters (Foldseek). The total and the non-redundant cluster counts are now given together.

*"We performed structural clustering…" In the M&M, MmSeqs2 is mentioned for PSC and DSC, not Foldseek.*

This error has been corrected. The attribution is now consistent throughout Results, Methods, and the deposit README: MMseqs2 performs sequence clustering (PSC, DSC) and Foldseek performs structural clustering (PXC, DXC).

*"To assess the global impact" — briefly describe the protocol used to obtain bacterial and eukaryotic datasets in this section but also in M&M.*

Added in both places: superkingdom occupancy of H-groups (bacteria, eukaryota, archaea) was derived directly from ECOD v292 annotations, and the Swiss-Prot comparator is our own classification of Swiss-Prot proteins.

*The paragraph "To place this shift in context" is unclear… proposes using a single organism as a model but ultimately compares all archaea. Clarify the rationale.*

Rewritten to make the logic explicit: the single organism (*M. jannaschii*, historically the only archaeon with a fully classified proteome) is the *starting baseline*: it recovers 542 shared and 13 archaea-specific H-groups; all archaea currently in ECOD raise these to 746/34; and our full classification raises them to 1,326/39. The point is that apparent archaeal fold scarcity is largely a sampling artifact, progressively resolved as sampling broadens from one organism to the full pan-archaeal set.

*…precise difference between non expanded and expanded. Provide a precise definition. The schematics of workflow… might help.*

Defined at first use (and shown in Fig 1A): the **non-expanded** set is the 71,866 proteins with a pre-existing AFDB structure; the **expanded** set is the 52,209 proteins with no AFDB structure that we predicted de novo with AlphaFold3 (22,883 UniParc + 29,326 Prodigal).

*"the lineage with the most distinctive cell biology" — How do the authors estimate this? Other archaeal lineages also [are distinctive]. How could it be quantified?*

We agree the superlative was not quantitatively supported (indeed, by group-specific fold count Euryarchaeota-related exceeds Asgard). We have removed the "most distinctive" claim and instead characterize the Asgard contribution specifically and quantitatively (its share of the residual dark-matter candidates and its eukaryote-signature families) without the unsupported superlative.

*"More than double the Swiss-Prot" — Why compare specifically to Swiss-Prot?*

The comparison is now framed as the question motivating the subsequent analysis: whether the elevated archaeal orphan fraction reflects genuine novelty or the classification of deeply divergent sequences against a reference (Swiss-Prot) built predominantly from bacterial and eukaryotic structures. Swiss-Prot is identified as that manually curated, well-classified reference, making it the natural baseline for an orphan rate.

*Figure 4C: The link between the text and the figure is unclear.*

Tightened: the sentence introducing the 25,965 proteins that lack a high-confidence assignment now cites Fig 4C directly.

*Define briefly "sub-threshold" in the Results section.*

Defined at first use in Results: a "sub-threshold" match is a DPAM domain hit that falls below the 0.85 high-confidence probability cutoff.

*"Are currently under expert curation" — replace with "requires further curation".*

Changed as suggested.

*Figure 7B is not mentioned in the text.*

A direct in-text reference to Fig 7B (the three a+b two-layered candidate novel folds) has been added.

*Figure 7: Why do some proteins carry the "-like" suffix and others not? Is there a threshold or criterion?*

There is no numeric threshold. "-like" is ECOD's curatorial resemblance nomenclature, applied by expert curation and occurring at every level of the hierarchy (~18–21% of named X-, H-, and T-group names). The specific terms in Figure 7 are the verbatim ECOD group names ("Rossmann-like" is ECOD X-group 2003 and "Zincin-like" is ECOD X-group 2498) and we now cite the ECOD level and accession at each such use. Where we instead propose a tentative assignment for a dark domain, the manuscript states the supporting evidence explicitly (e.g., Pfam PF16827; HHpred score 98.51; DaliLite matches), keeping that distinct from ECOD naming.

*Figures 8A and 8B appear to be inverted.*

Corrected. Figure 8 has been rebuilt with the correct panel order and an untangled caption (see Reviewer 1, point 6).

*The paragraph "The archaeal fold repertoire in context" should precede the previous one for better logical flow.*

The paragraph order has been adjusted for the flow the reviewer describes.
