# Supplementary material for: Domain classification of archaeal proteomes reveals conserved fold repertoire
Source: PLoS Comput Biol. 2026 Sep 18;22(9):e1014188. doi: 10.1371/journal.pcbi.1014188 (PMC13607799; doi:10.1371/journal.pcbi.1014188)
Supplement: Peer review history

# Peer review: Decision Letter 0

**DOI:** 10.1371/journal.pcbi.1014188.r001

**Authors:** Nir Ben-Tal, Joanna Slusky

3 Jul 2026

PCOMPBIOL-D-26-00716

Domain classification of archaeal proteomes reveals conserved fold repertoire

PLOS Computational Biology

Dear Dr. Schaeffer,

Thank you for submitting your manuscript to PLOS Computational Biology. After careful consideration, we feel that it has merit but does not fully meet PLOS Computational Biology's publication criteria as it currently stands. Therefore, we invite you to submit a revised version of the manuscript that addresses the points raised during the review process.

Please submit your revised manuscript by Sep 02 2026 11:59PM. If you will need more time than this to complete your revisions, please reply to this message or contact the journal office at ploscompbiol@plos.org. When you're ready to submit your revision, log on to https://www.editorialmanager.com/pcompbiol/ and select the 'Submissions Needing Revision' folder to locate your manuscript file.

Please include the following items when submitting your revised manuscript:

* A letter that responds to each point raised by the editor and reviewer(s). You should upload this letter as a separate file labeled 'Response to Reviewers'. This file does not need to include responses to formatting updates and technical items listed in the 'Journal Requirements' section below.

* A marked-up copy of your manuscript that highlights changes made to the original version. You should upload this as a separate file labeled 'Revised Manuscript with Track Changes'.

* An unmarked version of your revised paper without tracked changes. You should upload this as a separate file labeled 'Manuscript'.

If you would like to make changes to your financial disclosure, competing interests statement, or data availability statement, please make these updates within the submission form at the time of resubmission. Guidelines for resubmitting your figure files are available below the reviewer comments at the end of this letter.

As the corresponding author, your ORCID iD is verified in the submission system and will appear in the published article. PLOS supports the use of ORCID, and we encourage all coauthors to register for an ORCID iD and use it as well. Please encourage your coauthors to verify their ORCID iD within the submission system before final acceptance, as unverified ORCID iDs will not appear in the published article. Only the individual author can complete the verification step; PLOS staff cannot verify ORCID iDs on behalf of authors.

We look forward to receiving your revised manuscript.

Kind regards,

Joanna Slusky, Ph.D.

Academic Editor

PLOS Computational Biology

Nir Ben-Tal

Section Editor

PLOS Computational Biology

**Additional Editor Comments (if provided):**

**Journal Requirements:**

If the reviewer comments include a recommendation to cite specific previously published works, please review and evaluate these publications to determine whether they are relevant and should be cited. There is no requirement to cite these works unless the editor has indicated otherwise.

1) Please ensure that the CRediT author contributions listed for every co-author are completed accurately and in full.

At this stage, the following Authors/Authors require contributions: R. Dustin Schaeffer, Jimin Pei, Rui Guo, Jing Zhang, Kirill Medvedev, Qian Cong, and Nick Grishin. Please ensure that the full contributions of each author are acknowledged in the "Add/Edit/Remove Authors" section of our submission form.

The list of CRediT author contributions may be found here: https://journals.plos.org/ploscompbiol/s/authorship#loc-author-contributions

2) We ask that a manuscript source file is provided at Revision. Please upload your manuscript file as a .doc, .docx, .rtf or .tex. If you are providing a .tex file, please upload it under the item type u2018LaTeX Source Fileu2019 and leave your .pdf version as the item type u2018Manuscriptu2019.

3) Please provide an Author Summary. This should appear in your manuscript between the Abstract (if applicable) and the Introduction, and should be 150-200 words long. The aim should be to make your findings accessible to a wide audience that includes both scientists and non-scientists. Sample summaries can be found on our website under Submission Guidelines:

https://journals.plos.org/ploscompbiol/s/submission-guidelines#loc-parts-of-a-submission

4) We noticed that you used the phrase 'data not shown' in the manuscript. We do not allow these references, as the PLOS data access policy requires that all data be either published with the manuscript or made available in a publicly accessible database. Please amend the supplementary material to include the referenced data or remove the references.

5) Please upload all main figures as separate Figure files in .tif or .eps format. For more information about how to convert and format your figure files please see our guidelines:

https://journals.plos.org/ploscompbiol/s/figures

6) Please upload a copy of Figure Fig 1-8 which you refer to in your text on pages 9, 10, 11, 14, 15, 16, 19, and 20. Or, if the figure is no longer to be included as part of the submission please remove all reference to it within the text.

7) We have noticed that you have uploaded Supporting Information files, but you have not included a list of legends. Please add a full list of legends for your Supporting Information files after the references list.

8) Your current Financial Disclosure states, "The author(s) received no specific funding for this work." However, your funding information on the submission form indicates that you receive funding "GM147367, 1K99AI180984-01A1, DBI 2224128,I-1505,I-2095-20220331".Please indicate by return email the full and correct funding information for your study and confirm the order in which funding contributions should appear. Please be sure to indicate whether the funders played any role in the study design, data collection and analysis, decision to publish, or preparation of the manuscript.

9) We have amended your Competing Interest statement to comply with journal style. We kindly ask that you double check the statement and let us know if anything is incorrect.

**Reviewers' comments:**

Reviewer's Responses to Questions

**Comments to the Authors:**

**Please note here if the review is uploaded as an attachment.**

Reviewer #1: Summary Statement

In "Domain classification of archaeal proteomes reveals conserved fold repertoire," the authors provide comprehensive coverage of the sequence and structural universe of archaeal proteomes. They demonstrate that the protein fold repertoire is broadly conserved across all domains of life, settling the debate that structural novelty isn't the primary frontier.

Major/Minor Concerns

1. Clarification of Abstract Phrasing

I am confused by the abstract sentence: "40% of known structural diversity within a single domain of life." Does this imply that this single archaeal dataset encompasses 40% of the diversity found across all structural domains? Please clarify.

2. Structure Quality Metrics

On page 7, the text refers to proteins that "have structure quality metrics." Please specify which exact metrics (e.g., pLDDT, PAE) or thresholds were utilized to evaluate model confidence.

3. Phylogenetic Tree of Protein Classes

A phylogenetic tree of the 6 main classes of archaeal proteins would significantly enhance the reader's grasp of the evolutionary trajectory of these architectures.

4. Distinction of Asgard-Specific Distributions

On page 12, the authors note: "The distinction between Asgard-specific and pan-archaeal distribution has implications that are beyond the scope of this study but merit investigation..." A brief hint about what type of distinction was observed is highly warranted.

5. Methods Reference for Topology

On the same page, ensure you directly point the reader to the Methods section for explicit definitions of terms like "Partial Domain" or "simple topology."

6. Figure 8 Caption Discrepancies

The caption for Figure 8 does not make sense. Panel (A) appears to refer to structures in (B) and vice versa, and some structures are omitted entirely. It is unclear what the reader should be looking for in display (B); the labels require untangling.

7. Evaluation of Disordered Proteins

On page 15, the text notes that sequences "are predominantly disordered or poorly predicted." Since these proteins might hide structural novelty, disordered proteins or regions could be explicitly evaluated using sequence-based disorder predictors.

8. Woese vs two domain?

This analysis should inform the two-domain model, and I am disappointed that the authors do not share their insight on this question, especially given its apparent relevance.

9. Data accessibility and structure. The authors should provide a clearer description, possibly in the supplemental materials, of how the data are structured for readers who wish to use it. Specifically, is the dataset searchable? Are bulk download options available?

Overall, the paper is informative, well written, and provides in depth analysis of the sequence to structure anatomy of proteins.

Reviewer #2: The manuscript by Schaeffer et al. presents a thorough analysis of the archaeal protein domain and fold repertoire. The authors first identify new protein folds in archaeal genomes, noting that most are not truly novel due to sensitivity limitations. Comparative analysis with other domains of life reveals that most folds are shared across all three domains. While many newly identified folds result from low-quality predictions, the high-confidence ones are primarily associated with Asgards. Finally, the authors describe archaeal-specific folds, focusing on newly identified and two with distinct distribution patterns.

This study addresses a highly relevant topic, as 1) Archaea remain the least studied domain of life, offering a huge reservoir of uncharacterized proteins, and 2) Their role as ancestors of eukaryotes makes them key to understanding eukaryotic origins. Although the study does not yield groundbreaking findings, mapping the archaeal fold landscape is a valuable contribution to our understanding of life’s evolutionary history. The text is well-written and clear. However, I have identified several issues, particularly regarding the definition of terms and the logical flow of datasets, which is difficult to follow. Additionally, the authors should provide supplementary data, including all identifiers, domains, and the collection of new folds, to enhance the study’s impact for the community. Detailed suggestions and comments below.

General Comments

- The absence of line numbers complicates the review process.

- A global schematic summarizing the workflow of different datasets used in the study is missing. Such a figure would help readers navigate the various datasets (from initial database to the dark matter) presented across multiple figures and clarify the authors’ original contributions.

- The results should be compiled into supplementary material, including at least identifiers, associated characteristics (organism, Pfam domains, detected folds, H-group/X-group, Foldseek/MMseqs2 clusters (PSC, PXC, DSC, DXC), average pLDDT scores, origin of the structure). Additionally, a collection of predicted structures with unknown folds would be highly valuable. Providing these resources would significantly enhance the article’s impact, as they would enable further exploration by the community.

Specific Comments

- "Proteins were drawn" Specify "protein structures".

- "From the AFDB" Clarify "from AlphaFold2 (AF2)".

- Define terms such as pLDDT, DPAM judge, T-group, etc…

- "DPAM assigned 204,758" Does this refer to the total number of redundant domains? What about non-redundant domains?

- “We performed structural clustering…” In the M&M, MmSeqs2 is mentioned for PSC and DSC, not Foldeek.

- "To assess the global impact" Briefly describe the protocol used to obtain bacterial and eukaryotic datasets in this section but also in M&M.

- The paragraph "To place this shift in context" is unclear. The authors initially propose using a single organism as a model but ultimately compare all archaea. Clarify the rationale.

- I am not sure to understand what is the precise difference between non expanded and expanded. Provide a precise definition. The schematics of workflow of different datasets might help.

- “the lineage with the most distinctive cell biology” How do the authors estimate this statement? I agree that Asgards exhibit some special features but other archaeal lineages also do. How could it be quantified?

- "More than double the Swiss-Prot" Why compare specifically to Swiss-Prot?

- Figure 4C: The link between the text and the figure is unclear.

- Define briefly "sub-threshold" in the Results section.

- "Are currently under expert curation" replace with "requires further curation".

- Figure 7B is not mentioned in the text.

- Figure 7: Why do some proteins include the "-like" suffix while others do not? Is there a threshold or criterion?

- Figures 8A and 8B appear to be inverted.

- The paragraph "The archaeal fold repertoire in context" should precede the previous one for better logical flow.

**********

**Have the authors made all data and (if applicable) computational code underlying the findings in their manuscript fully available?**

The PLOS Data policy requires authors to make all data and code underlying the findings described in their manuscript fully available without restriction, with rare exception (please refer to the Data Availability Statement in the manuscript PDF file). The data and code should be provided as part of the manuscript or its supporting information, or deposited to a public repository. For example, in addition to summary statistics, the data points behind means, medians and variance measures should be available. If there are restrictions on publicly sharing data or code —e.g. participant privacy or use of data from a third party—those must be specified.

Reviewer #1: Yes

Reviewer #2: **No:** The results should be compiled into supplementary material

**********

PLOS authors have the option to publish the peer review history of their article (what does this mean?). If published, this will include your full peer review and any attached files.

If you choose “no”, your identity will remain anonymous but your review may still be made public.

**Do you want your identity to be public for this peer review?**  For information about this choice, including consent withdrawal, please see our Privacy Policy.

Reviewer #1: No

Reviewer #2: No

[NOTE: If reviewer comments were submitted as an attachment file, they will be attached to this email and accessible via the submission site. Please log into your account, locate the manuscript record, and check for the action link "View Attachments". If this link does not appear, there are no attachment files.]

**Figure resubmission:**

While revising your submission, we strongly recommend that you use PLOS’s NAAS tool (https://ngplosjournals.pagemajik.ai/artanalysis) to test your figure files. NAAS can convert your figure files to the TIFF file type and meet basic requirements (such as print size, resolution), or provide you with a report on issues that do not meet our requirements and that NAAS cannot fix.

After uploading your figures to PLOS’s NAAS tool - https://ngplosjournals.pagemajik.ai/artanalysis, NAAS will process the files provided and display the results in the "Uploaded Files" section of the page as the processing is complete. If the uploaded figures meet our requirements (or NAAS is able to fix the files to meet our requirements), the figure will be marked as "fixed" above. If NAAS is unable to fix the files, a red "failed" label will appear above. When NAAS has confirmed that the figure files meet our requirements, please download the file via the download option, and include these NAAS processed figure files when submitting your revised manuscript.

**Reproducibility:**

To enhance the reproducibility of your results, we recommend that authors of applicable studies deposit laboratory protocols in protocols.io, where a protocol can be assigned its own identifier (DOI) such that it can be cited independently in the future. Additionally, PLOS ONE offers an option to publish peer-reviewed clinical study protocols. Read more information on sharing protocols at https://plos.org/protocols?utm_medium=editorial-email&utm_source=authorletters&utm_campaign=protocols

# Author comment: Author response to Decision Letter 1

**DOI:** 10.1371/journal.pcbi.1014188.r002

29 Jul 2026

Attachment Submitted filename: Review Response - Archaea.docx

# Peer review: Decision Letter 1

**DOI:** 10.1371/journal.pcbi.1014188.r003

**Authors:** Nir Ben-Tal, Joanna Slusky

28 Aug 2026

PCOMPBIOL-D-26-00716R1

Domain classification of archaeal proteomes reveals conserved fold repertoire

PLOS Computational Biology

Dear Dr. Schaeffer,

Thank you for submitting your manuscript to PLOS Computational Biology. After careful consideration, we feel that it has merit but does not fully meet PLOS Computational Biology's publication criteria as it currently stands. Therefore, we invite you to submit a revised version of the manuscript that addresses the points raised during the review process.

Please submit your revised manuscript by Oct 28 2026 11:59PM. If you will need more time than this to complete your revisions, please reply to this message or contact the journal office at ploscompbiol@plos.org. When you're ready to submit your revision, log on to https://www.editorialmanager.com/pcompbiol/ and select the 'Submissions Needing Revision' folder to locate your manuscript file.

Please include the following items when submitting your revised manuscript:

* A letter that responds to each point raised by the editor and reviewer(s). You should upload this letter as a separate file labeled 'Response to Reviewers'. This file does not need to include responses to formatting updates and technical items listed in the 'Journal Requirements' section below.

* A marked-up copy of your manuscript that highlights changes made to the original version. You should upload this as a separate file labeled 'Revised Manuscript with Track Changes'.

* An unmarked version of your revised paper without tracked changes. You should upload this as a separate file labeled 'Manuscript'.

If you would like to make changes to your financial disclosure, competing interests statement, or data availability statement, please make these updates within the submission form at the time of resubmission. Guidelines for resubmitting your figure files are available below the reviewer comments at the end of this letter.

As the corresponding author, your ORCID iD is verified in the submission system and will appear in the published article. PLOS supports the use of ORCID, and we encourage all coauthors to register for an ORCID iD and use it as well. Please encourage your coauthors to verify their ORCID iD within the submission system before final acceptance, as unverified ORCID iDs will not appear in the published article. Only the individual author can complete the verification step; PLOS staff cannot verify ORCID iDs on behalf of authors.

We look forward to receiving your revised manuscript.

Kind regards,

Joanna Slusky, Ph.D.

Academic Editor

PLOS Computational Biology

Nir Ben-Tal

Section Editor

PLOS Computational Biology

**Additional Editor Comments (if provided):**

Before this manuscript can be accepted please ensure that the supplement is uploaded.

**Journal Requirements:**

If the reviewer comments include a recommendation to cite specific previously published works, please review and evaluate these publications to determine whether they are relevant and should be cited. There is no requirement to cite these works unless the editor has indicated otherwise.

1) Your current Financial Disclosure states, "This work was supported by grants from the National Institute of General Medical Sciences (GM147367 to R.D.S. and GM160468 to Q.C.; https://www.nigms.nih.gov/home), the National Institute of Allergy and Infectious Diseases (1K99AI180984-01A1 to J.Z.; https://www.niaid.nih.gov/), and the Welch Foundation (I-1505 to N.V.G.; I-2270-20260402 to Q.C.; https://welch1.org/). Computational resources were provided by NSF ACCESS (allocations MED230034 and MED240004; https://access-ci.org/) and TACC Lonestar6 (allocations MCB24018, MCB23014, and MCB26005; https://tacc.utexas.edu/). The funders had no role in study design, data collection and analysis, decision to publish, or preparation of the manuscript.".However, your funding information on the submission form not indicates "Computational resources were provided by NSF ACCESS (allocations MED230034 and MED240004; https://access-ci.org/) and TACC Lonestar6 (allocations MCB24018, MCB23014, and MCB26005; https://tacc.utexas.edu/)" that you receive funding. Please indicate by return email the full and correct funding information for your study and confirm the order in which funding contributions should appear. Please be sure to indicate whether the funders played any role in the study design, data collection and analysis, decision to publish, or preparation of the manuscript.

**Reviewers' comments:**

Reviewer's Responses to Questions

**Comments to the Authors:**

**Please note here if the review is uploaded as an attachment.**

Reviewer #1: The authors have satisfactorily addressed all of my concerns, and revisions have strengthened the manuscript.

Reviewer #2: The authors considered most of my comments and modified the manuscript accordingly. I am satisfied with this new version. The only issue that remains is that the link to access to the novel folds and the master table does not work.

**********

**Have the authors made all data and (if applicable) computational code underlying the findings in their manuscript fully available?**

The PLOS Data policy requires authors to make all data and code underlying the findings described in their manuscript fully available without restriction, with rare exception (please refer to the Data Availability Statement in the manuscript PDF file). The data and code should be provided as part of the manuscript or its supporting information, or deposited to a public repository. For example, in addition to summary statistics, the data points behind means, medians and variance measures should be available. If there are restrictions on publicly sharing data or code —e.g. participant privacy or use of data from a third party—those must be specified.

Reviewer #1: Yes

Reviewer #2: **No:** The link to access to the novel folds and the master table should work.

**********

PLOS authors have the option to publish the peer review history of their article (what does this mean?). If published, this will include your full peer review and any attached files.

If you choose “no”, your identity will remain anonymous but your review may still be made public.

**Do you want your identity to be public for this peer review?**  For information about this choice, including consent withdrawal, please see our Privacy Policy.

Reviewer #1: No

Reviewer #2: No

[NOTE: If reviewer comments were submitted as an attachment file, they will be attached to this email and accessible via the submission site. Please log into your account, locate the manuscript record, and check for the action link "View Attachments". If this link does not appear, there are no attachment files.]

**Figure resubmission:**

While revising your submission, we strongly recommend that you use PLOS’s NAAS tool (https://ngplosjournals.pagemajik.ai/artanalysis) to test your figure files. NAAS can convert your figure files to the TIFF file type and meet basic requirements (such as print size, resolution), or provide you with a report on issues that do not meet our requirements and that NAAS cannot fix.

After uploading your figures to PLOS’s NAAS tool - https://ngplosjournals.pagemajik.ai/artanalysis, NAAS will process the files provided and display the results in the "Uploaded Files" section of the page as the processing is complete. If the uploaded figures meet our requirements (or NAAS is able to fix the files to meet our requirements), the figure will be marked as "fixed" above. If NAAS is unable to fix the files, a red "failed" label will appear above. When NAAS has confirmed that the figure files meet our requirements, please download the file via the download option, and include these NAAS processed figure files when submitting your revised manuscript.

**Reproducibility:**

To enhance the reproducibility of your results, we recommend that authors of applicable studies deposit laboratory protocols in protocols.io, where a protocol can be assigned its own identifier (DOI) such that it can be cited independently in the future. Additionally, PLOS ONE offers an option to publish peer-reviewed clinical study protocols. Read more information on sharing protocols at https://plos.org/protocols?utm_medium=editorial-email&utm_source=authorletters&utm_campaign=protocols

# Author comment: Author response to Decision Letter 2

**DOI:** 10.1371/journal.pcbi.1014188.r004

1 Sep 2026

Attachment Submitted filename: Response to reviewers- archaea R1.docx

# Editor report: Decision Letter 2

**DOI:** 10.1371/journal.pcbi.1014188.r005

**Authors:** Nir Ben-Tal, Joanna Slusky

3 Sep 2026

Dear Dr. Schaeffer,

We are pleased to inform you that your manuscript 'Domain classification of archaeal proteomes reveals conserved fold repertoire' has been provisionally accepted for publication in PLOS Computational Biology.

Before your manuscript can be formally accepted you will need to complete some formatting changes, which you will receive in a follow up email. A member of our team will be in touch with a set of requests.

Please note that your manuscript will not be scheduled for publication until you have made the required changes, so a swift response is appreciated.

IMPORTANT: The editorial review process is now complete. PLOS will only permit corrections to spelling, formatting or significant scientific errors from this point onwards. Requests for major changes, or any which affect the scientific understanding of your work, will cause delays to the publication date of your manuscript.

Should you, your institution's press office or the journal office choose to press release your paper, you will automatically be opted out of early publication. We ask that you notify us now if you or your institution is planning to press release the article. All press must be co-ordinated with PLOS.

Thank you again for supporting Open Access publishing; we are looking forward to publishing your work in PLOS Computational Biology.

Best regards,

Joanna Slusky, Ph.D.

Academic Editor

PLOS Computational Biology

Nir Ben-Tal

Section Editor

PLOS Computational Biology

***********************************************************

# Editor report: Acceptance letter

**DOI:** 10.1371/journal.pcbi.1014188.r006

**Authors:** Nir Ben-Tal, Joanna Slusky

PCOMPBIOL-D-26-00716R2

Domain classification of archaeal proteomes reveals conserved fold repertoire

Dear Dr Schaeffer,

I am pleased to inform you that your manuscript has been formally accepted for publication in PLOS Computational Biology. Your manuscript is now with our production department and you will be notified of the publication date in due course.

The corresponding author will soon be receiving a typeset proof for review, to ensure errors have not been introduced during production. Please review the PDF proof of your manuscript carefully, as this is the last chance to correct any errors. Please note that major changes, or those which affect the scientific understanding of the work, will likely cause delays to the publication date of your manuscript.

Soon after your final files are uploaded, unless you have opted out, the early version of your manuscript will be published online. The date of the early version will be your article's publication date. The final article will be published to the same URL, and all versions of the paper will be accessible to readers.

For Research, Software, and Methods articles, you will receive an invoice from PLOS for your publication fee after your manuscript has reached the completed accept phase. If you receive an email requesting payment before acceptance or for any other service, this may be a phishing scheme. Learn how to identify phishing emails and protect your accounts at https://explore.plos.org/phishing.

Thank you again for supporting PLOS Computational Biology and open-access publishing. We are looking forward to publishing your work!

With kind regards,

Janani Seenivasan

PLOS Computational Biology | Carlyle House, Carlyle Road, Cambridge CB4 3DN | United Kingdom ploscompbiol@plos.org | Phone +44 (0) 1223-442824 | ploscompbiol.org | @PLOSCompBiol
